# New Insights on the Role of the pLMST6 Plasmid in *Listeria monocytogenes* Biocide Tolerance and Virulence

**DOI:** 10.3389/fmicb.2019.01538

**Published:** 2019-07-09

**Authors:** Alexandra C. Kropac, Athmanya K. Eshwar, Roger Stephan, Taurai Tasara

**Affiliations:** Institute for Food Safety and Hygiene, Vetsuisse Faculty, University of Zurich, Zurich, Switzerland

**Keywords:** *Listeria monocytogenes*, biocide, benzalkonium chloride, *emrC*, pLMST6, plasmid, quaternary ammonium compound

## Abstract

*Listeria monocytogenes* the causative agent of listeriosis is an important public health concern and food safety challenge. Increased tolerance of this bacterium to benzalkonium chloride (BC), an antibacterial agent widely used in industrial settings, is a growing issue. Plasmid pLMST6 harboring the gene of the multidrug efflux pump protein EmrC has been recently linked to enhanced BC tolerance and meningitis due to *L. monocytogenes* ST6 strains. In this study, occurrence and contribution of this plasmid to BC tolerance was examined using PCR, plasmid curing and transformation, RT-qPCR and proteome analysis, respectively. Furthermore, the substrate specificity of the pLMST6 associated EmrC efflux pump and the impact of the plasmid on *L. monocytogenes* virulence were investigated. pLMST6 was detected in 7 (1.6%) of 439 *L. monocytogenes* strains isolated from different sources. A phenotypic role of this plasmid in conferring increased BC tolerance was confirmed by showing that plasmid cure increases BC susceptibility whereas plasmid complementation and transformation increased BC tolerance in different *L. monocytogenes* genetic backgrounds and *L. innocua*. RT-qPCR showed that BC stress exposure strongly induces the expression of mRNAs associated with pLMST6 genes for EmrC and a TetR transcription regulator. A full proteome analysis in a plasmid harboring *L. monocytogenes* strain revealed that the pLMST6 encoded putative TetR family transcription regulator protein is the most upregulated protein in response to BC stress exposure. An investigation into the EmrC efflux pump’s substrate spectrum showed that while pLMST6 confers increased tolerance to other quaternary ammonium compounds (QACs) based disinfectants it has no impact on the sensitivity of *L. monocytogenes* to non-QAC disinfectants as well as on antibiotics such as ampicillin, tetracycline and gentamicin. A reduction in the survival of zebrafish embryos infected with pLMST6 plasmid harboring *L. monocytogenes* strains was observed when compared with plasmid cured variants of the same strains suggesting that some pLMST6 harbored genes might contribute to increased virulence capacity. Overall these results confirm the phenotypic contribution of pLMST6 plasmid in promoting and dissemination of BC tolerance in *L. monocytogenes* as well as provide new insights on different molecular levels of pLMST6 associated genes in response to BC stress.

## Introduction

Listeriosis is a severe foodborne infection caused by *Listeria monocytogenes*, a gram-positive, facultative intracellular bacterium occurring ubiquitously in soil and water. Due to its ability to survive and grow under various environmental stresses and persist over extended periods of time, this pathogen is considered a growing concern in food industry (Fox et al., 2011). In 2017 the European Food Safety Authority reported a listeriosis incidence of 0.48 cases per 100,000 population, among which 227 deaths were registered (EFSA, 2018). In patients with weakened immune systems consumption of food contaminated with *L. monocytogenes* can lead to severe illness and high mortality as well as cause abortions in pregnant women (Maertens de Noordhout et al., 2014).

*Listeria monocytogenes* comprises of thirteen serotypes that are subdivided into four main evolutionary genetic lineages and several multilocus sequence typing (MLST) clones, which show variable distribution in food products and processing environments as well as among human and animal clinical listeriosis cases (Orsi et al., 2011; Haase et al., 2014; Maury et al., 2016). In food processing plants quaternary ammonium compounds (QACs) such as benzalkonium chloride (BC), benzethonium chloride (BZC) and cetyltrimethylammonium bromide (CTAB) are frequently used as disinfectants (Morente et al., 2013). However, tolerance to broad spectrum disinfectants is a growing concern and increased tolerance to BC has been reported among *L. monocytogenes* strains (Aase et al., 2000; Mereghetti et al., 2000; Dutta et al., 2013; Müller et al., 2013; Xu et al., 2014; Meier et al., 2017). BC acts on the cytoplasmic membrane permeability in microorganisms causing cytosolic leakage and degradation of proteins and nucleic acids (McDonnell and Russell, 1999). Exposure to sublethal BC concentrations for bacteria in food associated environments can lead to development of BC tolerance in pathogens (To et al., 2002). The existence of environmental niches in food processing plants that are difficult to reach for disinfectants leading to exposure of targeted bacterial pathogens such as *L. monocytogenes* to sublethal concentrations is therefore a major concern.

Bacterial tolerance to QAC can be either an intrinsic property as for example biofilm formation or acquired through several molecular and physiological mechanisms (McDonnell and Russell, 1999). Non-specific acquired tolerance mechanisms include changes in cell wall and cell membrane that prevent entrance of the disinfectant agent into the cell. Specific tolerance mechanism can for example include efflux pump genes located on genetic material acquired by horizontal gene transfer.

Until now a number of different efflux pump systems leading to increased BC tolerance in *L. monocytogenes* have been discovered (Elhanafi et al., 2010; Müller et al., 2013; Kovacevic et al., 2015). These include QacH, a transporter encoded by genes located on the Tn6188 transposon and the benzalkonium chloride resistance cassette BcrABC encoded by genes located on a plasmid (pLM80) or on the chromosome (Elhanafi et al., 2010; Müller et al., 2013). EmrB is a transmembrane protein responsible for the efflux of drugs across the outer membrane leading to multidrug resistance that was firstly identified in *Escherichia coli* (Lomovskaya and Lewis, 1992; Paulsen et al., 1996). Homologs of this proton motive force dependent multidrug efflux protein have now also been detected in gram positive bacteria including EmrE described in *L. monocytogenes* (Kovacevic et al., 2015). Recently it was shown that the plasmid pLMST6, carrying a gene encoding a quaternary ammonium efflux protein homologous to EmrB called EmrC, does not only confer increased BC tolerance but is also associated with meningitis linked to ST6 *L. monocytogenes* strains (Kremer et al., 2017). The plasmid pLMST6 was shown to be 4268 bp in size and comprises of seven open reading frames, encoding two putative transcriptional regulators (Tet/AcR and CopG), a putative recombination mobilization protein (Mob/Rec), a plasmid replicase (RepB), two hypothetical proteins and the quaternary ammonium efflux protein, EmrC. Meanwhile besides possibly enhancing virulence through multidrug resistance, some of the efflux pumps system were also shown to have physiological roles that influence virulence in *L. monocytogenes* by secreting cyclic-di-AMP that triggers the production of type I interferons in infected macrophages (Schwartz et al., 2012; Zeevi et al., 2013). A potential association between efflux pumps carrying plasmids and their contribution to virulence, however, still needs to be elucidated.

RT-qPCR and proteome analysis are both important tools that can be used to study gene expression responses in microorganisms exposed to stress conditions. While RT-qPCR provides information on the regulation of targeted genes at mRNA level, the full proteome analysis approach on the other hand provides global insights at protein level about the actual cellular processes that are modulated in response to stress exposure and takes into consideration both posttranscriptional regulation as well as posttranslational modifications of gene expression.

The aim of this study was to determine the occurrence of pLMST6 plasmid among *L. monocytogenes* strains previously isolated from various sources as well as to determine the contribution of the plasmid to BC tolerance at phenotypic and molecular levels, including pLMST6 regulation during BC stress response using RT-qPCR and LC-MS/MS based proteome analysis. In addition, the plasmid associated EmrC efflux pump substrate specificity and impact of pLMST6 plasmid on *L. monocytogenes* virulence were investigated.

## Materials and Methods

### Ethics Statement

This study was carried out in accordance with the principles and recommendations of the Ordinance on laboratory animal husbandry, the production of genetically modified animals and the methods of animal experimentation; Animal Experimentation Ordinance (SR 455.163, April 12, 2010), Swiss Federal Food Safety and Veterinary Office (FSVO/BLV). The maximum age reached by the zebrafish embryos during experimentation was 5 dpf (days post fertilization) for which no license was required from the cantonal veterinary office in Switzerland, since embryos had not yet reached free feeding stage. Husbandry and breeding of the adult zebrafishes were performed at the Institute for Molecular Life Sciences, University of Zurich, Zurich, Switzerland. All animal protocols used were in compliance with internationally recognized and with Swiss legal ethical guidelines for the use of fish in biomedical research and experiments were approved by the local authorities (Veterinäramt Zürich Tierhaltungsnummer 150).

### Bacterial Strains and Growth Conditions

A total of 439 *L. monocytogenes* strains were examined in this study that are listed in Table 1 and Supplementary Table S1. Strains from Switzerland (*n* = 242) were collected through the Swiss National Reference Centre for Enteropathogenic Bacteria and Listeria (NENT). Finnish strains (*n* = 197) were collected through the Department of Food Hygiene and Environmental Health of the Faculty of Veterinary Medicine at the University of Helsinki. Bacteria stocks of each strain were stored at −80°C in brain heart infusion (BHI; Oxoid, Pratteln, Switzerland) broth containing 20% glycerol (Sigma-Aldrich, Buchs, Switzerland). Strains were streaked out on blood agar plates (Difco, Columbia blood agar base, 5% sheep blood, Oxoid, Switzerland) or BHI agar plates. To prepare inoculum, single colonies picked from each strain were cultured for 16 h in BHI broth at 37°C and 200 rpm.

**Table 1:** Bacterial strains used in this study.

Strain-ID^1^	Country of origin	Source	Serotype	Sequence type	Clonal complex	pLMST6 presence
N12-1667	CH	Environment	1/2a	403	403	Yes
N11-1547	CH	Human blood	1/2a	403	403	Yes
N12-0935	CH	Human blood	1/2a	403	403	Yes
N13-0094	CH	Human blood	1/2a	403	403	Yes
N12-2082	CH	Human blood	1/2a	8	8	Yes
LT30E	FIN	Vegetable	1/2a	8	8	Yes
N05_195	CH	Meat	1/2a	31	31	Yes
N11-2553	CH	Human blood	1/2a	403	403	Absent
N11-1546	CH	Human blood	1/2a	8	8	Absent
Lm3136	CH	Human blood	1/2a	18	18	Absent
N16-0044	CH	Human blood	4b	6	6	Absent
LL195	CH	Human blood	4b	1	1	Absent
N13-1054	CH	Human blood	4b	2	2	Absent
EGDe^2^	GB	Guinea pig	1/2a	35	9	Absent
JF5051^3^	SE		6a			Absent

*^1^For the rest of the strains see Supplementary Table S1. ^2^EGDe strain ATCC BAA-679 (WT), originally isolated from guinea pig (Murray et al., 1926). ^3^Listeria innocua type strain ATCC 33090, purchased from the Culture Collection University of Götenborg, internal collection.*

### pLMST6 Presence in *L. monocytogenes*

To assess for pLMST6 presence, conventional PCR was used to screen 439 *L. monocytogenes* strains, which were previously isolated between 1999 and 2016 from human listeriosis cases, food products and processing environments (Supplementary Table S1). Single colonies picked from blood agar plates were suspended in 200 μl of lysis buffer (20 mM tris pH 8.0. 2 mM EDTA, 1.2% triton X-100), incubated 10 min at 90°C and briefly centrifuged (10 s at 9 000 *g*). PCR was performed in 50 μl volume reactions containing Go Taq G2 Green Master Mix (Promega, Switzerland; 25 μl), DNA templates (5 μl) derived from boiling of single bacterial colonies and primers (0.5 μM) targeting pLMST6 plasmid associated *emrC* (387 bp) and replication protein (*rep*, 318 bp) genes (see Table 2). PCR cycling conditions applied were as follows: 2 min at 94°C; 36 cycles at 94°C for 15 s, 50°C for 30 s and 72°C for 30 s; and a final extension at 72°C for 5 min. Negative controls to which no DNA was added and N11-2553 known to contain no plasmid were included in all PCR reactions. DNA purified from N12-0935 using the GenElute^TM^ Bacterial Genomic DNA Kit (Sigma-Aldrich, Buchs, Switzerland) was used as a positive control. The presence and size of PCR amplicons were determined with agarose gel electrophoresis (1% agarose gel stained with ethidium bromide; 50 V for 90 min). PCR primers for *emrC* were designed to specifically amplify the pLMST6 associated gene for EmrC to the exclusion of the closely related *qacH* sharing a 74.59% sequence identity with *emrC* that may also be found in other *L. monocytogenes* strains.

**Table 2:** Primers used in this study for PCR detection of tolerance genes and RT-qPCR.

Primer	Genetic target	Sequence (5′–3′)	Efficiency (%)	References
EmrC fw	*emrC*	TTA TTC CAT TTT ATT ACT GGC AAT G	95.8	This study
EmrC rv		CGT ATT TAT ATT TAA CAC TAG CCA		
RpA fw	*rep*	ATG GCT AAA GAC AAA GCA AG		This study
RpA rv		CTT TTG ATT CAT GTG TTA AAT ACA G		
tetR fw	*tetR*	AGT GGC GAT AGC TCA AA	88.0	This study
tetR rv		GGA ATG GTA CAA GAC TGG A		
16S rRNA fw	16S rRNA	CTT CCG CAA TGG ACG AAA GT	94.0	Michel, 2012
16S rRNA rv		CTC ATC GTT TAC GGC GTG		

### Determination of Minimum Inhibitory Concentration (MIC)

The minimum inhibitory concentrations (MICs) of BC, BZC, CTAB, chlorhexidine and Halades PE were determined through optical density-based growth curve analysis using the Synergy HT OD reader (BioTek Instruments GmbH, Lucerne, Switzerland). Strains were streaked out on blood agar plates and incubated for 24 h at 37°C to yield single colonies. Single colonies from each strain were inoculated into 10 ml BHI broth and incubated overnight (16–18 h at 37°C and 200 rpm). A total of 10^7^ CFU/ml cultures from each strain were added to 96 well microtiter plates in BHI supplemented with increasing concentrations of BC (0, 3, 4, 5, 6, 7, and 8 μg/ml, Sigma-Aldrich, Buchs, Switzerland), BZC (0, 3, 4, and 5 μg/ml, Sigma-Aldrich, Buchs, Switzerland), CTAB (0, 2, 3, 4, and 5 μg/ml), chlorhexidine (0, 0.0002, 0.0003, and 0.0004%, Sigma-Aldrich, Buchs, Switzerland), and peracetic acid based Halades PE (0, 0.25, 0.30, and 0.35%, Halag Chemie AG, Switzerland). The plates were incubated for 20 h at 37°C with shaking and growth was monitored at 30-minute intervals in a Synergy HT OD reader (BioTek Instruments GmbH, Lucerne, Switzerland). The MIC for the disinfectant agents was determined as the lowest concentration that prevented growth (threshold OD_600_ ≤ 0.1) after 20 h of incubation at 37°C. The MICs for gentamicin (0.016–256 μg/ml), ampicillin (0.016–256 μg/ml) and tetracycline (0.016–256 μg/ml) were determined by the use of E-test strips (BioMérieux, France). E-test inoculum (McFarland 0.5, BioMérieux, France) preparation and plating, strip application and subsequent MIC determinations were carried out in accordance with the manufacturer’s instructions. The MICs determined as the lowest antibiotic concentration preventing growth were read on Mueller-Hinton agar plates (MHA; Oxoid, Pratteln, Switzerland) or blood agar plates after 24 h of incubation at 37°C.

### pLMST6 Plasmid Curing and Transformation

To determine whether BC tolerance is pLMST6 associated, the plasmid was cured from four plasmid-carrying strains by heat treatment (Elhanafi et al., 2010). The pLMST6 plasmid containing N11-1547, N12-0935, N12-2082, and N13-0094 strains were inoculated into BHI and incubated overnight (16–18 h) at 40°C. Resulting primary cultures were subcultured in BHI and incubated overnight at 40°C. The procedure was repeated for over 14 generations after which the final broth cultures were plated out on BHI agar plates and incubated overnight at 37°C. Single colonies were picked from the different strains and re-plated on BC BHI (10 μg/ml BC) agar plates. The colonies derived from plasmid cured strains were identified by the failure to grow on BC-BHI agar while able to grow on the BHI agar plates. Plasmid loss in such derivatives was confirmed by *emrC* and *repA* PCR analysis as described above. Colonies lacking *emrC* and *rep* PCR product bands were identified as plasmid cured variants (PCVs) from each strain. To verify the observed BC sensitivity in the PCVs, complementary pLMST6-carrying transformants (T) were obtained through transformation of PCVs of strains N12-0935 and N12-2082. In addition, the pLMST6 plasmid was transformed into six wild type plasmid naive *L. monocytogenes* (N1546, Lm3136, N16-0044, LL195, N13-1054 and EGDe) and one *L. innocua* (JF5051) strain (see Table 1). Plasmid transformations were carried out by electroporation as previously described (Monk et al., 2008) and transformed strains were selected by incubating for 24–72 h at 37°C on BC BHI plates and confirmed by PCR analysis with *emrC* and *rep* primers as described above (Table 2).

### Reverse Transcription Quantitative PCR Analysis

Reverse transcription quantitative-PCR (RT-qPCR) was used to determine the impact of BC stress exposure on mRNA levels of pLMST6 associated *emrC* and *tetR* genes. Overnight cultures of pLMST6 plasmid harboring strain *L. monocytogenes* N12-0935 were inoculated into 10 ml of normal and BC (3 μg/ml) supplemented BHI and the cultures were grown until the stationary phase (16 h at 37°C and 200 rpm). One milliliter stationary phase cultures standardized to OD_600_ of 1 (10^9^ CFU/ml) were harvested in RNA protect Bacteria reagent (Qiagen, Hombrechtikon, Switzerland) and resuspended in 0.5 ml RNeasy Plus Mini Kit (Qiagen, Hombrechtikon, Switzerland) lysis buffer. The samples were lysed (2 × 6500 rpm for 60 s with 1 min ice cooling between the steps) using the MagNA Lyser instrument (Roche Molecular Diagnostics, Risch-Rotkreuz, Switzerland). Total RNA isolation was performed using RNeasy Plus Mini kit (Qiagen, Hombrechtikon, Switzerland) according to the manufacturer’s instructions including an on-column DNAse I (Qiagen, Hombrechtikon, Switzerland) digestion step. RNA was eluted in 50 μL of RNAse free water. Purified RNA samples were quantified using the Nanodrop instrument (Thermo Scientific, United States) and its quality was assessed with the RNA 6000 Pico Chip kit and a BioAnalyzer (Agilent Technologies, United States). RNA integrity numbers (RINs) above 8.0 were achieved in all samples. Reverse transcription was performed using the Quantitect Reverse Transcription Kit (Qiagen, Hombrechtikon, Switzerland). 480 ng (*tetR*) and 1000 (*emrC*) ng of RNA per sample were converted into cDNA in 20 μl. Residual DNA contamination in the RNA samples was ruled out by inclusion of no RT controls in the analysis. Real-time PCR reactions were performed with primers listed in Table 2 on a Light Cycler 480 instrument (Roche Molecular Diagnostics, Risch-Rotkreuz, Switzerland). The total reaction volume of 20 μl contained 27.8 ng (*emrC*) and 1.2 ng (*tetR*) cDNA, 0.5 μM of each primer and the LightCycler^R^ 480 SYBR Green I master mix (Roche Molecular Diagnostics, Penzberg, Germany). The real-time PCR cycling conditions were as follows: Preincubation at 95°C for 5 min, amplification 10 s at 95°C; 20 s at 50°C; 20 s at 72°C; 1 s at 78°C with a single fluorescent measurement, melting curve (65–97°C at 2.2°C/s and a continuous fluorescent measurement) followed by a cooling down phase to 40°C. Dilution series of N12-0935 genomic DNA-based standard curves were used to determine the efficiencies of target gene amplification by real-time PCR. The target gene amplifications used in this study had PCR efficiencies ranging from 88.0 to 95.8% (Table 2). Each sample was analyzed in triplicate on three independent occasions and the results presented reflect the means and standard deviations. Relative cDNA quantification was performed using the Light Cycler 480 Relative Quantification Software (Roche Molecular Diagnostics). The mRNA amounts were normalized using 16S rRNA as a reference gene (Tasara and Stephan, 2007).

### Proteome Sample Preparation and Measurement

Overnight precultures of strain N12-0935 prepared in duplicate as described above were inoculated into 10 ml of normal (2%) and BC (4%; 3 μg/ml) supplemented BHI broth and incubated 18 h at 37°C and 200 rpm. The resulting secondary cultures were standardized to OD_600_ of 1 (10^9^ CFU/ml) using PBS. 10 ml of each sample were centrifuged at 4000 g at 4°C for 10 min. Bacterial pellets were resuspended in 300 μl denature buffer (Sample Preparation Kit ^©^BiognoSYS AG, Schlieren, Switzerland). Pooled duplicate samples were mechanically lysed (2 × 6500 rpm for 60 s with 1 min ice cooling between the steps) using the MagNA Lyser instrument (Roche Molecular Diagnostics, Rotkreuz Switzerland). One microliter of benzonase^^®^^ nuclease (Sigma-Aldrich, Buchs, Switzerland) was added and the samples were incubated at room temperature for 30 min and centrifuged 5 min at 5000 *g*. Supernatants were transferred into new 1.5 ml Eppendorf tubes and protein concentration was measured using a Coomassie (Bradford) Protein Assay Kit (Thermo Scientific, Switzerland) and the NanoDrop spectrophotometer (Witec AG, Switzerland). Bovine serum albumin (BSA; Thermo Scientific, Switzerland) was used as a standard. The samples were then prepared according to the cell culture sample preparation procedure protocol of the Sample Preparation Kit (BiognoSYS AG, Schlieren, Switzerland) according to the manufacturer’s instructions. One microliter of trypsin (Sequencing Grade Modified Trypsin 20 μg, Promega, Switzerland) was added to each sample and incubated overnight at 37°C with shaking at 600 rpm. C18 clean up procedure was conducted to purify and concentrate the peptides in the samples. C18 spin columns (MicroSpin^TM^ Columns, The Nest Group, Inc., United States) were conditioned by adding 200 μl of 100% methanol (Auer Brittmann soulié AG, Switzerland) and centrifugation for 60 s at 200 g. Columns were cleaned by adding 200 μl of C18 cleaning solution (80% Acetonitril (Thermo Scientific, Switzerland); 0.1% Trifluoroacetic acid (TFA; Fluca BioChemica, Switzerland), in distilled water) and centrifugation for 60 s at 200 *g*. Then 200 μl of washing solution (1% Acetonitril; 0.1% TFA, in distilled water) was applied three times and centrifuged as above. Overnight incubated samples were centrifuged 16000 *g* for 60 s. Resulting supernatants were loaded onto the spin columns and centrifuged (200 *g* for 60 s). The flow-through was collected and re-loaded to the column and centrifuged to improve protein yield. The columns were washed three times by adding 200 μl of the C18 washing solution and centrifugation for 60 s at 200 *g*. The samples were eluted with 350 μl of C18 solution (50% Acetonitril, 0.1% TFA, in distilled water) and vacuum dried (Concentrator 5301, Eppendorf, Germany) at 60°C for 100 min.

Peptides were resuspended in 20 μl LC solvent A (1% acetonitrile, 0.1% formic acid). The final peptide concentration was determined using a UV/VIS Spectrometer (SpectroSTAR nano, BMG Biotech, Germany). For the LC-MS/MS (shotgun) measurements, 2 μg of peptides from each sample were injected to a C18 column (Dr. Maisch ReproSil Pur, 1.9 μm particle size, 120 Å pore size; 75 μm inner diameter, 40 cm length, New Objective) packed by Biognosys in-house on a Thermo Scientific Easy nLC 1000 nano-liquid chromatography system connected to a Thermo Scientific Q Exactive mass spectrometer equipped with a nanospray FLEX-electrospray source. LC solvents were A: 1% acetonitrile in water with 0.1% formic acid; B: 15% acetonitrile in water with 0.1% formic acid. The non-linear LC gradient was 1–52% solvent B in 120 min followed by 52–90% B in 0.1 min and 90% B for 10 min. A modified TOP12 method was used (Kelstrup et al., 2012). The mass spectrometric data was analyzed using the SpectroMine 1.0.18206.1.26682 search engine (Biognosys AG, Schlieren, Switzerland) the false discovery rate on PSM, peptide and protein level was set to 1%. A specific protein fasta file was developed based on the nucleotide sequence of *L. monocytogenes* strain N12-0935 genome (accession number CP038642) and the plasmid pLMST6 (accession number CP038643) that have been deposited in GenBank at the National Center for Biotechnology Information (NCBI) database. To sequence this strain genomic DNA was extracted and purified using the GenElute Bacterial Genomic DNA Kit (Sigma, Buchs, Switzerland). Genome sequencing was subsequently conducted at ChunLab Seoul National University using Pacific Biosciences single-molecule real-time sequencing technology (SMRT) chemistry. Sequences generated were assembled *de novo* using the SMRT Analysis v2.3.0 software and the Hierarchical Genome Assembly Process (HGAP_3) workflow. Genome annotation was carried out through the NCBI Prokaryotic Genome Annotation Pipeline. N12-0935 genome analysis including pLMST6 plasmid search and comparison were conducted in CLC genomics Workbench (Qiagen, Prismet, Denmark) and using BLASTn and BLASTp in the NCBI platform (blast.ncbi.nlm.nih.gov/Blast.cgi). For HRM LC-MS/MS measurements, 2 μg of peptides were injected onto an identical LC-MS set up. A DIA method with one full range survey scan and 18 DIA windows was used. Samples were measured in three technical replicates and analyzed with Spectronaut version 12.0.20491.19.

### Virulence Analysis Using Zebrafish Larvae

In order to assess for the potential virulence impact of the pLMST6 plasmid, *L. monocytogenes* strains N12-0935 and N12-2082 that naturally harbor pLMST6 and their respective PCVs were compared. The virulence assays were conducted through microinjection infection of 2 dpf zebrafish embryos as previously described (Eshwar et al., 2017). Briefly, bacterial suspension (100 CFU) was injected into blood circulation via the caudal vein. After the injection, the embryos were allowed to recover in fresh E3 medium (5 mM NaCl, 0.17 mM KCl, 0.33 mM CaCl_2_, and 0.33 mM MgSO_4_) for 15 min. The embryos were incubated at 28°C and observed for survival under a stereomicroscope twice a day over 3 days.

### Statistical Analysis

Microsoft Excel 2013 was used to generate growth curves and Kaplan-Meier plots, as well as to perform statistical analysis of the BC MIC and gene expression data. Statistical significance of differences in BC MICs between the plasmid naive and pLMST6 transformed variants of the different *L. monocytogenes* strains were evaluated using the Wilcoxon rank-sum test. To evaluate for the statistical significances in differences of *emrC* and *tetR* mRNA levels between *L. monocytogenes* N12-0935 cells grown under BC stress and controls grown in BHI, one-way analysis of variance (ANOVA) was used. Prior to analysis the relative expression ratios determined for the two genes were first normalized through log_10_ conversion. Fold inductions of *emrC* and *tetR* mRNA levels in response to BC stress were calculated by dividing the log converted relative mRNA levels of each gene under BC stress with those in the BHI grown controls. The spectronaut software 12.0.20491.19 (Biognosys AG, Schlieren, Switzerland) was used for the analysis of data from the proteome experiments. In all cases of statistical analysis differences were considered significant when *P*-values were ≤0.05 and *q*-values (multiple testing corrected *p*-values; proteome analysis) ≤0.05.

## Results

### Occurrence of the pLMST6 Plasmid in *L. monocytogenes* Strains

In the present study we developed a strategy to screen for pLMST6 presence in *L. monocytogenes* strains involving two PCR assays to detect pLMST6 plasmid located genes *emrC* and *rep*, encoding for the EmrC efflux pump and the putative plasmid replication initiator protein RepA, respectively. Based on this strategy an analysis of 439 *L. monocytogenes* strains previously isolated from diverse sources (clinical, food production environments and food products) in Switzerland and Finland detected pLMST6 in seven strains (CH: 6/242, 2.5%; FIN: 1/197, 0.5%) indicating a 1.6% prevalence of this plasmid in this strain collection. The isolates harboring the plasmid were all serotype 1/2a strains and belonged to MLST clonal complexes CC403 (ST403; *n* = 4), CC8 (ST8; *n* = 2) and CC31 (ST31; *n* = 1), and were isolated from raw meat, vegetables, food production environments and human listeriosis cases (Table 1). A phenotypic analysis confirmed that all the pLMST6 harboring strains had a reduced susceptibility to BC compared to other strains lacking the plasmid. The BC MIC among the pLMST6 strains ranged from 20 to 30 μg/ml in contrast to plasmid free *L. monocytogenes* strains that showed an MIC range of 7.5–10 μg/ml BC as assayed on MHA plates.

### pLMST6 Confers Increased BC Tolerance in *L. monocytogenes* and *L. innocua* Strains

**FIGURE 1:**
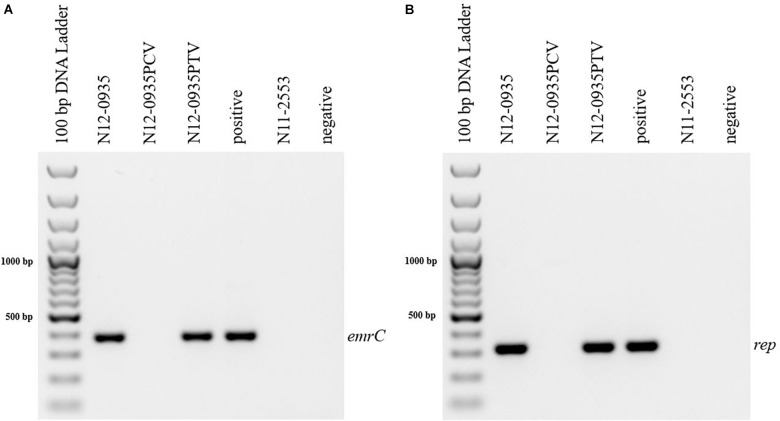
Detection of pLMST6 plasmid genes in *L. monocytogenes*. Primers targeting *emrC* (387 bp) **(A)** and *rep* (318 bp) **(B)** genes were used. PCR amplicons were determined with agarose gel electrophoresis. Negative controls to which water or DNA of *L. monocytogenes* strain N11-2553 (lacks the pLMST6 plasmid) was added were included. DNA purified from N12-0935 (natural strain harboring pLMST6) was used as a positive control.

**FIGURE 2:**
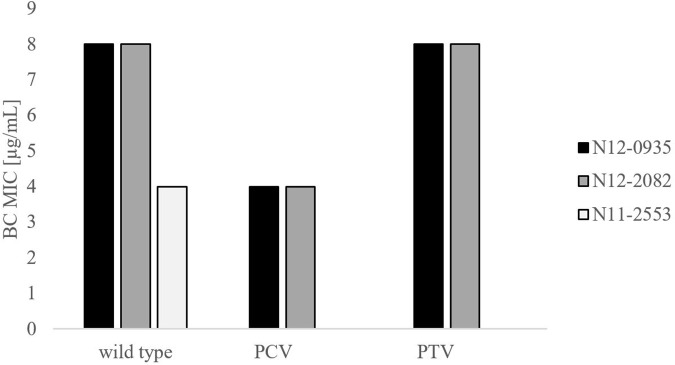
Phenotypic impact of pLMST6 plasmid on BC tolerance in *L. monocytogenes* N12-0935 and N12-2082 strains. MICs were determined in BHI broth supplemented with increasing concentrations of BC and incubated for 20 h at 37°C. N12-0935 and N12-2082 represent the wild type strains naturally harboring pLMST6, PCVs the respective plasmid cured variants and PTV the respective complemented strains in which pLMST6 was reintroduced by transformation of PCVs. Data was obtained from three independent biological replicates.

**FIGURE 3:**
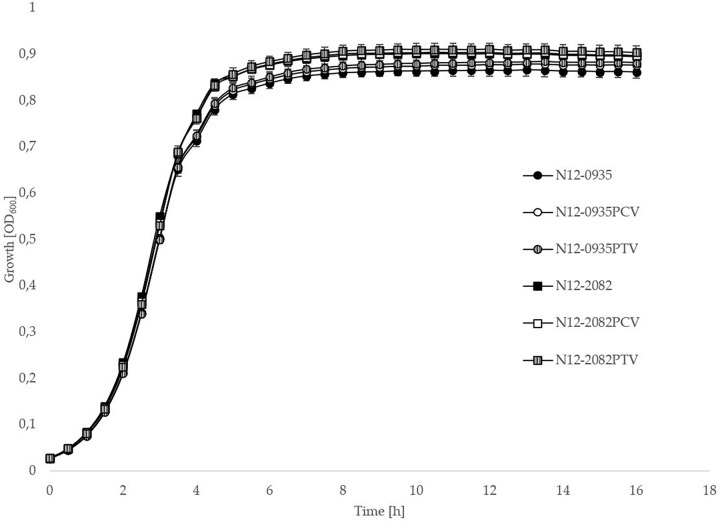
pLMST6 plasmid does not have a significant impact on *L. monocytogenes* fitness during growth in BHI. Optical density-based growth curves of natural plasmid harbouring, PCVs and PTVs of *L. monocytogenes* strains N12-0935 and N12-2082 in BHI cultures incubated for 18 h at 37°C. Data was obtained from three independent biological replicates.

In order to confirm the phenotypic contribution of the pLMST6 plasmid harboring the *emrC* gene to increased BC tolerance, plasmid cured (PCV) and plasmid transformed (PTV) variants were created in two *L. monocytogenes* strains N12-0935 (CC403) and N12-2082 (CC8) that also naturally harbor the pLMST6 plasmid. PCR analysis for *emrC* and *rep* genes was used to confirm creation of the PCVs and PTVs in the genetic background of these two strains (Figure 1). BC sensitivity analysis conducted on BHI plates supplemented with 10 μg/ml BC showed that while the plasmid carrying wild type parental N12-0935 and N12-2082 strains grew, their PCVs were unable to grow on such plates (data not shown). On the other hand, growth on such BHI-BC plates could be restored for the N12-0935PTV and N12-2082PTV strains that were generated by re-transforming the PCVs (N12-0935PCV and N12-2082PCV) of both strains with the pLMST6 plasmid. Further assessment of BC susceptibility in BC supplemented BHI broths similarly showed that plasmid curing decreased the BC MICs for N12-0935PCV and N12-2082PCV to 4 μg/ml compared to 8 μg/ml BC observed in their respective wild type parental strains that naturally harbor the pLMST6 plasmid. The BC MICs determined for N12-0935PCV and N12-2082PCV were similar to that of pLMST6 plasmid free *L. monocytogenes* strain N11-2553 that was used as a negative control. Meanwhile re-transforming pLMST6 into the plasmid cured strains (N12-0935PCV and N12-2082PCV) in N12-0935PTV and N12-0282PTV strains restored the BC MIC to levels that are identical (8 μg/ml) to their plasmid harboring parental strains thus showing that complementation of the BC tolerance phenotypes involves pLMST6 plasmid associated genetic elements (Figure 2). A kinetic growth comparison showed similar growth behavior in natural plasmid harboring N12-0935 and N12-2082 parental wild type strains as well as in their PCVs and PTVs in BHI at 37°C indicating that there was no fitness reduction associated with the presence of pLMST6 plasmid without BC stress under the experimental conditions of our study (Figure 3).

**FIGURE 4:**
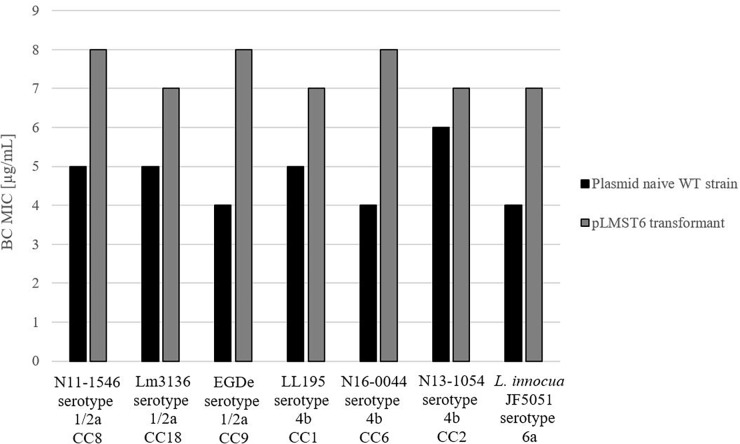
pLMST6 plasmid confers increased BC tolerance in different *Listeria* genetic backgrounds. BC MICs determined for different *L. monocytogenes* and *L. innocua* strains in BHI broth. Transformants of N11-1546 (CC8), Lm3136 (CC18), EGDe (CC9), LL195 (CC1), N16-0044 (CC6), N13-1054 (CC2) and *L. innocua* showed an increased BC MICs ranging from 17 to 100% increase compared to untransformed parent strains. In all cases difference between parental and transformed strains were statistically significant (*p* ≤ 0.05; pairwise comparison Wilcoxon rank-sum test). Data was obtained from three independent biological replicates and performed in duplicates.

To further investigate pLMST6 contribution to BC tolerance in context of different *Listeria* genetic backgrounds and other *Listeria* species, the pLMST6 plasmid was transformed into *L. innocua* and six other *L. monocytogenes* strains derived from genetic lineage I serotype 1/2a (EGDe, Lm3136 and N11-1546) and genetic lineage II serotype 4b (LL195, N16-0044 and N13-1054) representing MLST clonal complexes CC8, CC18, CC35, CC1, CC2, and CC6. The selected *L. monocytogenes* strains included four clinical isolates from previous listeriosis outbreaks (Lm3136, LL195, N11-1546 and N16-0044), as well as one food isolate (N13-1054) and the *L. monocytogenes* reference strain EGDe. *L. innocua* strain JF5051 could also be successfully transformed with pLMST6 leading to increased BC tolerance indicating the potential of this plasmid to be transformed between different *Listeria* species. BC MICs in all the six *L. monocytogenes* pLMST6 transformants generated were also increased relative to their corresponding plasmid naive parental strains (Figure 4). The extent to which BC MIC increased upon pLMST6 transformation among the listeria strains was, however, strain dependent, with the EGDe (CC9) and N16-0044 (CC6) showing the highest magnitude of MIC increase (100%), followed by *L. innocua* (75%), N11-1546 (CC8; 60%), LL195 (CC1; 40%) and Lm3136 (CC18; 40%). The lowest magnitude of MIC increase upon pLMST6 transformation was detected with N13-1054 (CC2; 17%). Compared to the other tested strains, N13-1054 exhibited a significantly higher BC MIC prior to transformation. The higher BC tolerance phenotype in this strain is due to the fact that it also harbors the Tn6188 associated BC resistance gene *qacH*. Overall these results showed that while pLMST6 confers increased BC tolerance the magnitude of phenotypic tolerance conferred is also dependent on the *Listeria* strain genetic background.

### Transcripts Encoded by pLMST6 Plasmid Associated Genes Are Induced in Response to BC Stress Exposure of *L. monocytogenes*

**FIGURE 5:**
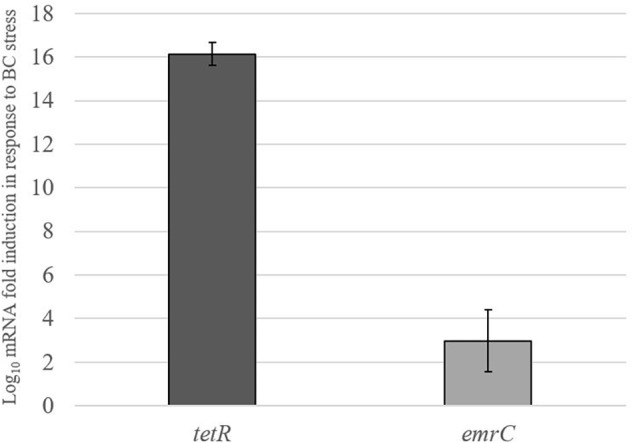
pLMST6 plasmid encoded *tetR* and *emrC* mRNAs are induced in response to BC stress. Levels of *tetR* and *emrC* mRNAs in *L. monocytogenes* N12-0935 cells grown in regular and BC (3 μg/ml) supplemented BHI were quantified by RT-qPCR. Relative quantities (RQ) of the mRNAs were normalized to 16S rRNA. Data represent the means (bars) and standard deviations (±1 SD) from three independent biological experiments. The *tetR* and *emrC* mRNA levels were significantly higher (*p* ≤ 0.01 based on one-way ANOVA and *post hoc* Tukey HSD test) in N12-0935 cells grown under BC stress in BHI compared to BHI grown controls.

Real-time qRT-PCR was used to examine the impact of BC stress exposure on the expression of pLMST6 plasmid associated genes *tetR* and *emrC* at transcript level in *L. monocytogenes* N12-0935 that naturally harbors this plasmid. Genes examined encode for a putative TetR (*tetR*) family transcriptional regulator and the EmrC (*emrC*) efflux pump protein. The level of mRNAs encoded by these two genes was compared between N12-0935 cells grown to early stationary phase (16 h at 150 rpm and 37°C) under BC stress (3 μg/ml BC) in BHI and controls that had been similarly grown but without BC stress. Growth under BC stress was associated with a significant induction of both *tetR* and *emrC* mRNA levels compared to controls (Figure 5). Relative to controls grown without BC stress the N12-0935 cells grown under BC stress in BHI showed 16 and 3-log fold inductions in *tetR* and *emrC* mRNA levels, respectively.

### Proteome Analysis Identifies pLMST6 Encoded Proteins Amongst Proteins Upregulated in Response to BC Stress Exposure in Plasmid Harboring *L. monocytogenes* Cells

**FIGURE 6:**
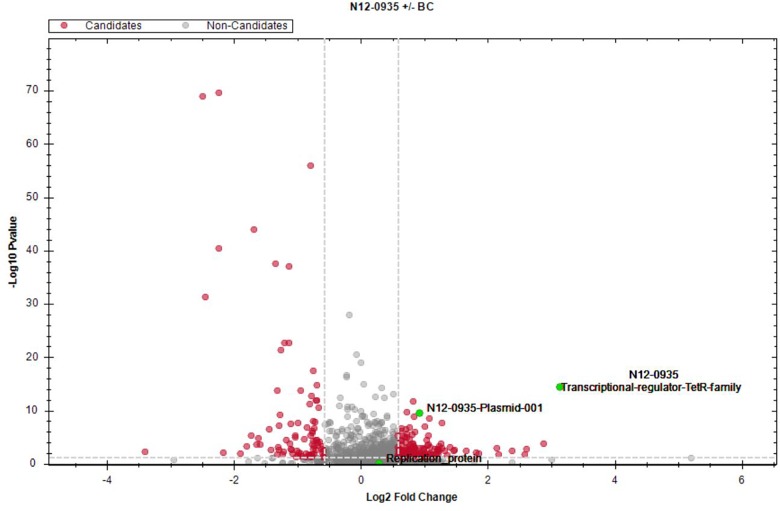
Impact of BC stress on protein expression in *L. monocytogenes* N12-0935. A volcano plot of protein abundance (fold change versus *t*-test *p* value) showing proteins that were regulated in cells of *L. monocytogenes* N12-0935 that were grown under BC stress (BHI plus 3 μg/ml BC) compared to controls similarly grown in normal BHI without BC. Red dots indicate significantly up- and down-regulated proteins (*q* ≤ 0.05, average log_2_ ratio ≥0.58, gray dashed lines). Green dots denote three pLMST6-derived upregulated proteins, TetR (*q*-value 1.03E-13, average log_2_ ratio 3.13), N12-0935_Plasmid-011 (*q*-value 3.14E-9, average log_2_ ratio 0.93) and N12-0935_Replication-protein (*q*-value 0.31, average log_2_ ratio 0.29).

To assess for proteins that are regulated in response to BC stress a full proteome analysis of *L. monocytogenes* strain N12-0935 was conducted by mass spectrometry comparing protein expression levels between N12-0935 cells grown to stationary phase under BC stress (3 μg/ml BC) with controls similar grown but without BC stress in BHI broth. A total of 1369 proteins representing 47.4% (1369/2887) of the targeted proteins in N12-0935 including the pLMST6 plasmid encoded proteins were identified. Two hundred and forty-two of the detected proteins were found to be regulated in response to BC stress exposure (Supplementary Table S2). These included 157 and 85 proteins that were significantly upregulated and downregulated, respectively (Figure 6; *q* ≤ 0.05 and average log_2_ ratio ≥0.58). Overall, this full proteome analysis showed, that a complex stress response network is triggered in response to BC stress exposure that includes the upregulation of various proteins involved in cell wall and membrane modifications, transport, metabolism and gene regulation. On the other hand, BC stressed cells seemed to downregulate peptidoglycan bound proteins, including numerous autolysins and virulence factors. But for the purposes of this study we only focused on pLMST6 encoded proteins identified and their regulation in response to BC stress exposure. Out of the six pLMST6 encoded proteins, three could be detected under our experimental conditions (Table 3). Only N12-0935_Plasmid-001 (*q*-value 3.14E-09, average log_2_ ratio 0.93), representing a putative transcriptional regulator, and N12-0935_Transcriptional-regulator-TetR-family (*q*-value 1.03E-13, average log_2_ ratio 3.13) representing TetR were significantly upregulated. Interestingly, TetR was the most upregulated protein in response to BC stress exposure detected in this study. Although the N12-0935_Replication-protein was marginally upregulated the results were not statistically significant (*q*-value 0.31, average log_2_ ratio 0.29). Meanwhile proteins for the efflux pump EmrC, N12-0935_Plasmid-002 and N12-0935_Plasmid-recombination-enzyme were not detected.

**Table 3:** Plasmid encoded proteins identified in *L. monocytogenes* N12-0935 based on full proteome analysis.

Protein-ID	Locus tag^1^	Function^2^	Log_2_ fold change	*Q* value	Status
N12-0935_Plasmid-001	E5D16_15145	Putative transcriptional regulator	0.93	3.14E-09	Significant upregulated
N12-0935_Transcriptional-regulator-TetR-family	E5D16_15165	Putative transcriptional regulator	3.13	1.03E-13	Significant upregulated
N12-0935_Replication-protein	E5D16_15150	Putative plasmid replicase	0.29	0.31	Not significant regulated

*^1^Locus tags as deposited in GenBank at NCBI database (accession number CP038643). ^2^Protein functional assignment is according to Kremer et al. (2017).*

### pLMST6 Confers Increased Resistance to QACs but Not to Non-QAC Disinfectants and Antibiotics

The substrate spectrum of the EmrC efflux pump was determined against a selection of QAC based (BZC and CTAB) and non-QAC based disinfectants (chlorhexidine and peracetic acid) and antibiotics (ampicillin, gentamicin and tetracycline). BZC, CTAB, chlorhexidine and peracetic acid are commonly used disinfectants that *L. monocytogenes* might encounter in food associated environments. Ampicillin and gentamicin are currently the antibiotic treatment of choice for listeriosis in humans. Tetracycline is one therapeutic option for listeriosis treatment in cattle and a common acquired resistance mechanism against this antibiotic involves efflux pumps. MICs for these compounds were determined in plasmid harboring wild type parental strains *L. monocytogenes* N12-0935 and N12-2082, PCVs (N12-0935PCV and N12-2082PCV) generated from these strains, as well as pLMST6 PTVs (N12-0935PTV and N12-2082PTV), that were generated by re-transformation of the PCVs with the pLMST6 plasmid. Compared to plasmid harboring wild type parental strains, the PCVs of both strains N12-0935PCV and N12-2082PCV, showed an increased susceptibility to BZC (5 vs. 3 μg/ml) and CTAB (3 vs. 2 μg/ml) upon loss of the pLMST6 plasmid. The BZC (5 μg/ml) and CTAB (3 μg/ml) MICs were, however, restored to parental wild type strain levels upon re-transformation of the PCVs with the pLMST6 plasmid in PTVs N12-0935PTV and N12-2082PTV. On the other hand, there were no MIC differences observed between the parental wild type strains and the PCVs (N12-0935PCV and N12-2082PCV) and PTVs (N12-0935PTV and N12-2082PTV) when exposed to the disinfectants, chlorhexidine and peracetic acid (Table 4). Ampicillin, gentamicin and tetracycline MICs of the parental strains, PCVs and PTVs were all similar in organisms with (16 h pre-growth at 37°C in BHI plus 3 μg/ml BC) and without (16 h pre-growth in BHI at 37°C) prior preadaptation to BC stress. Overall our results thus showed that while the QACs BZC and CTAB are efflux substrates for the EmrC, this efflux pump system has no protective effect against other non-QAC based disinfectants and antibiotics as tested at least under experimental conditions of this study.

**Table 4:** The MICs of various disinfecting and antibiotic agents for *L. monocytogenes* strain N12-0935 and N12-2082, the plasmid cured variants and the pLMST6 transformants.

Substance:	MIC						
	BZC (μg/ml)	CTAB (μg/ml)	Chlorhexidine (%)	Halades PE (%)	Ampicillin^1^ (μg/ml)	Gentamicin^1^ (μg/ml)	Tetracycline (μg/ml)
N12-0935	5	3	0.0004	0.3	0.047	0.125	0.38
N12-0935PCV	3	2	0.0004	0.3	0.047	0.125	0.38
N12-0935PTV	5	3	0.0004	0.3	0.032	0.125	0.38
N12-2082	5	3	0.0004	0.25	0.125	0.125	0.25
N12-2082PCV	3	2	0.0004	0.25	0.125	0.094	0.25
N12-2082PTV	5	3	0.0004	0.25	0.125	0.125	0.25

*^1^MIC determination on MHA plates after 24 h incubation at 37°C.*

### Impact of the pLMST6 Plasmid on *L. monocytogenes* Virulence

**FIGURE 7:**
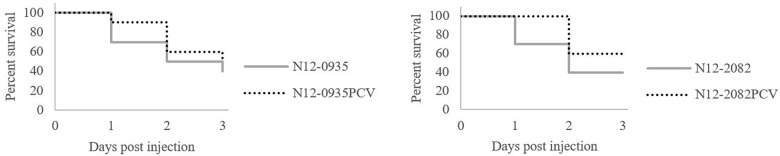
pLMST6 plasmid increases *L. monocytogenes* virulence. Kaplan-Meier plots depicting the survival trends of zebrafish embryos (*n* = 30 per *L. monocytogenes* strain) infected (100 CFU per embryo) with plasmid harboring wild type N12-0935 and N12-2082 strains and their PCVs during 72 h of incubation. Embryos infected with pLMST6 harboring wild type strain showed lower survival compared to those infected with PCVs. Data was obtained from three independent biological replicates. Survival curves and trends were significantly different between zebrafish embryos infected with plasmid harboring wild type strains and their respective PCVs (log rank test, *p* < 0.05).

To assess the impact of pLMST6 plasmid on *L. monocytogenes* virulence capacity we used a zebrafish embryo based multicellular *in vivo* infection model. Zebrafish embryos were infected through blood stream microinjection with pLMST6 plasmid harboring *L. monocytogenes* N12-0935 (CC403) and N12-0282 (CC8) strains and their PCVs. Post infection the embryos were monitored for survival at daily intervals over 3 days (72 hpi, hours post infection). As shown in Figure 7, a small but significant reduction in survival of zebrafish embryos was observed after infection with pLMST6 plasmid harboring variant compared to PCV for each of the two *L. monocytogenes* strains (Figure 7). Our observations thus indicate that there is an increase in *L. monocytogenes* virulence associated with the presence of some genes harbored on the pLMST6 plasmid that manifests as increased mortality of zebrafish embryos infected with plasmid carrying bacterial cells.

## Discussion

In our previous studies, we revealed upon the examination of a collection of *L. monocytogenes* strains isolated from humans, foods and food processing associated environments in Switzerland and Finland the existence of various BC tolerant strains that possible harbor yet unknown BC resistance genetic mechanisms (Meier et al., 2017). Such strains despite displaying relatively high BC tolerance levels (MICs greater than 10 μg/ml BC on MHA agar) lacked genes of known BC resistance efflux pump systems in *L. monocytogenes* such as *qacH*, *brcABC* and *emrE*. In a recent study, a novel plasmid named pLMST6, which carries a gene for EmrC has been associated with increased BC tolerance as well as reduced susceptibility to the antibiotics amoxicillin and gentamicin, and increased incidences of listerial meningitis linked to ST6 *L. monocytogenes* strains in the Netherlands (Kremer et al., 2017).

In order to examine for the distribution of such plasmids within our previous collection of *L. monocytogenes* strains whole genome sequencing was first applied in a BC tolerant strain N12-0935 that lacks previously known genes for BC tolerance efflux pump systems (accession number: CP038642 and CP038643). The presence of the pLMST6 plasmid in this strain was detected from genome sequence analysis and subsequently confirmed through PCR analysis. Comparing the N12-0935 pLMST6 plasmid (CP03864) and the previously sequenced pLMST6 plasmid (LT7326401) revealed that the two plasmids are highly conserved in sequence identity (99.91% identity based on 100% query coverage by discontiguous megablast). The pLMST6 previously sequenced by Kremer et al. (2017) is 4268 bp whereas the N12-0935 pLMST6 is 4265 bp. In both plasmids seven genes encoding two putative transcriptional regulators (TetR and CopG), a recombination protein, a plasmid replication protein (RepB), two hypothetical protein and EmrC are present. Almost all the proteins in the two plasmids share 100% amino acid identity except for the plasmid replication protein RepB, which shows 95% amino acid identity and differ by a single amino acid between the two plasmids. Prevalence of pLMST6 plasmid within our strain collection was subsequently determined through PCR analysis of 439 *L. monocytogenes* strains comprised of isolates recovered from animals, raw and processed foods, processing environments and human listeriosis patients. Strains investigated were dominated by serotypes 1/2a, 1/2b, 1/2c, and 4b making the collection representative of typical *L. monocytogenes* serotypes frequently detected in food processing environment and human clinical listeriosis cases (Orsi et al., 2011). The pLMST6 plasmid was overall detected in 1.6% (7/439) of the examined strains, while at individual country levels the plasmid prevalence was 2.5% (6/242; Switzerland) and 0.5% (1/197; Finland), respectively, among the examined *L. monocytogenes* strains. Strains that were detected to harbor pLMST6 plasmid included 43% of the BC tolerant strains previously described as lacking known BC resistance genes in our previous studies (Meier et al., 2017). Apart from Switzerland and Finland, pLMST6 plasmid presence in *L. monocytogenes* strains was so far also documented in the Netherlands where it was detected in 5% of the examined human listeriosis isolates (Kremer et al., 2017). The pLMST6 plasmid in our study was detected in serotype 1/2a ST403 (*n* = 4/7; 57.1%), ST8 (*n* = 2/7; 28.5%) and ST31 (*n* = 1/7; 1.4%) strains (see Table 1), whereas in the Netherlands the plasmid was found in ST6 (4b) and ST9 (1/2a) strains. In Switzerland both ST8 and ST403 represent *L. monocytogenes* strains that are frequently isolated from human listeriosis and food products (Ebner et al., 2015). The observations reported to date thus indicate pLMST6 is disseminated among different *L. monocytogenes* serotypes and genotypes. It might be tempting to speculate that pLMST6 presence within these serotype 1/2a and 4b strains could provide some selective advantage for the survival and dissemination of these serotypes in food processing environments where QACs such as BC are encountered. The pLMST6 harboring strains detected in our studies represent both infected humans as well as food and environment associated isolates, whereas those reported from the Netherlands were all from humans and associated with listeria meningitis cases that frequently led to poor clinical outcomes. In view of the Swiss pLMST6 harboring human isolates detected from our study there is insufficient clinical epidemiological information to determine the associated disease severity and final clinical outcome in the infected patients. On the other hand, pLMST6 in our study was more frequently detected among the Swiss CC403 strains and this observation might be indicative of early stages of clonal expansion of the plasmid within this genotype in Switzerland. However, future surveillance studies will be necessary to determine if this might indeed be the case. As in the previous observations, all the pLMST6 harboring isolates identified in our study showed reduced BC susceptibility compared to other strains that do not contain pLMST6 or other known BC resistance genetic elements.

In order to confirm the phenotypic association between pLMST6 plasmid presence and BC tolerance, PCVs and PTVs of two *L. monocytogenes* strains N12-0935 and N12-2082 were created. These two strains belong to MLST clonal complexes CC403 and CC8 and were isolated from two different human listeriosis cases. A phenotypic analysis conducted in these two strains confirmed that curing for the pLMST6 plasmid leads to loss of the BC tolerant phenotype whereas transformation with this plasmid into a plasmid free background completely restores the BC tolerance phenotype. In order to further assess pLMST6 contribution to BC tolerance in other *Listeria* genetic backgrounds, the pLMST6 plasmid was also transformed into *L. innocua* and six other *L. monocytogenes* strains representing genetic lineage I and clonal complexes CC8, CC9 and CC18, as well as genetic lineage II and clonal complexes CC1, CC2 and CC6. Besides *L. innocua* and the *L. monocytogenes* reference strain EGDe, the transformed strains included clinical isolates associated with previous Swiss listeriosis outbreaks including: strain LL195 (serotype 4b; CC1) that was responsible for 1983–1987 Vacherin Montd’or cheese listeriosis epidemic (Weinmaier et al., 2013); strain Lm3136 (serotype 1/2a; CC18) from the 2005 Tomme cheese listeriosis outbreak in northwest Switzerland (Bille et al., 2005); strain N11-1546 (serotype 1/2a; CC8) involved in the 2011 listeriosis outbreak associated with imported ham products (Hächler et al., 2013); and strain N16-0044 (serotype 4b; CC6) that caused the 2016 local listeriosis outbreak in southern Switzerland due to contaminated meat pâté (Althaus et al., 2017). In addition, strain N13-1054 a ST2 BC resistant isolate that harbors the gene for the BC efflux pump QacH was included to a assess the impact of pLMST6 transformation into an existing BC resistant genetic background. In all the different *L. monocytogenes* MLST genetic backgrounds tested as well as *L. innocua* the ability of the pLMST6 plasmid to increased BC tolerance could be confirmed since all the transformed strains displayed increased BC MICs compared to their corresponding plasmid naive parental strains. The highest increases in BC MICs associated with pLMST6 transformation were induced in CC9 and CC6 strains whereas there were modest increases observed for CC18 and CC1 strains suggesting that the extent to which pLMST6 genes contribute to BC tolerance also varies depending on the *L. monocytogenes* strain genetic background. In N13-1054, which already carries the *qacH* BC resistance there was a slight but significant further increase in BC MIC induced by pLMST6 transformation showing that the acquisition of this plasmid has an additive effect on reducing BC susceptibility when acquired in genetic backgrounds where another BC resistant efflux pump system is already present. Our results also demonstrated the potential horizontal transferability of this efflux pump harboring plasmid not only in different *L. monocytogenes* genetic backgrounds but also to different *Listeria* species. As *in vivo* horizontal interspecies plasmid transfer harboring drug-resistance determinants have also been observed in different other bacterial pathogens our observations suggest that transfer of BC tolerance through pLMST6 plasmid between *Listeria* strains is possible and may spread BC tolerance within food processing environment associated niches, which reinforces the growing concern in food safety (Tauxe et al., 1989; Boguslawska et al., 2009; Goren et al., 2010). Meanwhile previous studies have also shown that bacteria harboring plasmids in some cases had a significant longer lag phase compared to plasmid free strains indicating a fitness reduction (Smith and Bidochka, 1998). But kinetic growth comparisons of N12-0935 and N12-2082 (Figure 3) as well as all the other pLMST6 transformed strains generated in this study with their corresponding plasmid free parental strains (data not shown) showed similar growth behavior in presence or absence of pLMST6 as assessed in BHI at 37°C. An observation indicating that there is no significant fitness reduction in *L. monocytogenes* strains associated with the presence of the pLMST6 plasmid, which could be due to negligible metabolic stress of maintaining small plasmids or low plasmid copy numbers (Kües and Stahl, 1989; Valenzuela et al., 1996).

In order to garner further insights into whether pLMST6 plasmid associated genes and proteins are being activated in response to BC stress we also assessed the impact of BC exposure on the expression of transcripts and proteins encoded by the pLMST6 plasmid. At transcript level the impact of BC stress (BHI plus 3 μg/ml BC) on the expression of pLMST6 plasmid harbored genes for EmrC and the putative TetR family transcriptional regulator was examined using RT-qPCR. A significant induction of mRNAs encoded by both genes was observed in response to BC stress exposure of plasmid harboring strain N12-0935 indicating that there is indeed a strong transcriptional activation of these pLMST6 associated genes in response to BC stress exposure in this bacterium. It was shown, that the relationship between gene expression at transcript level and protein production is poorly correlated in bacteria since posttranscriptional regulations as well as posttranslational modifications may lead to altered results between transcriptome and proteome analysis (Kocharunchitt et al., 2012; Liu et al., 2016). To provide further molecular insights into the regulation of pLMST6 plasmid encoded proteins at proteome level a full proteome analysis of the BC stress response in *L. monocytogenes* N12-0935 was also conducted. In this case, two putative transcriptional regulators, one of which belongs to the TetR family transcriptional regulators and located upstream of the *emrC* efflux pump gene, were discovered among proteins that were significantly upregulated in response to BC stress. Our results therefore suggest important roles for these proteins in regulating the expression of pLMST6 genes including *emrC* during BC stress response within *L. monocytogenes* cells. While the function of these regulators remain to be investigated in the context of *L. monocytogenes*, it has been shown that some TetR family transcription regulators are involved in control multidrug efflux systems in other bacteria where such proteins mediate transcriptional repression (Ramos et al., 2005; Deng et al., 2013). It is thus possible that the induction observed for the putative TetR regulator is required for the controlled expression activation of the pLMST6 associated genes under BC stress. Overall our results thus provided both mRNA and protein level evidence confirming the molecular activation of pLMST6 genes and consequently their functions for BC stress responses in plasmid carrying *L. monocytogenes* cells.

Besides pLMST6 plasmid derived proteins, this study also revealed that 242 of 1369 proteins detected in strain N12-0935 were significantly regulated in response to BC stress (Supplementary Table S2). This included the upregulation of proteins involved in cell wall and membrane modifications, transport, metabolism and gene regulation as well as downregulation of peptidoglycan bound proteins, including numerous autolysins and virulence factors. Based on these results, a complex stress response protein network that includes pLMST6 associated proteins seems to be triggered in response to BC stress, which comprises of different molecular mechanisms whose phenotypic contribution to BC tolerance in *L. monocytogenes* remains to be investigated. Meanwhile we could not detect the EmrC protein using LC-MS/MS in the *L. monocytogenes* N12-0935 proteome fractions generated in this study. One possible explanation for this is that membrane associated proteins such as EmrC might not have been present in the generated proteome fractions since the sample preparation protocols used are not optimized for isolation of membrane associated proteins, which remains a common challenge for sample preparation in proteomic analysis (Chandramouli and Qian, 2009). As such sample preparation protocol adjusted to target membrane proteins could be used in future in combination with either LC-MS/MS or quantitative two-dimensional gel electrophoresis that include fluorescent labeling of the proteins to assess for the presence and upregulation of EmrC within membrane proteins of pLMST6 plasmid harboring *L. monocytogenes* cells exposed to BC stress.

To further investigate the disinfectant efflux substrate spectrum of the pLMST6 associated EmrC efflux pump, we showed that pLMST6 conferred increased tolerance to other QAC-based disinfectants such as BZC and CTAB. On the other hand, we found that pLMST6 could not confer increased tolerance against other widely used non-QAC based substances including broad spectrum antiseptic products such as chlorhexidine and a peracetic acid-based sanitizer investigated in this study. These results thus indicate that the EmrC associated pLMST6 plasmid seems to confer specific protection against QAC based disinfectants. Other studies confirmed that various efflux pump systems in *L. monocytogenes* were also QAC specific, as was the case for EmrE (sharing 52.34% amino acid identity with EmrC) which was discovered on a novel genomic island (LGI1) (Kovacevic et al., 2015) and QacH (sharing 74.59% amino acid identity with EmrC), a transporter located on the Tn6188 transposon (Müller et al., 2013). Additionally, it has also been described that *L. monocytogenes* isolates carrying the pLMST6 plasmid were associated with decreased susceptibility toward antibiotics such as amoxicillin and gentamicin (Kremer et al., 2017). In contrast, examination of our collection of pLMST6 PCVs and PTVs in the different *L. monocytogenes* genetic backgrounds as well as *L. innocua* revealed that the pLMST6 plasmid does not have an influence on ampicillin, gentamicin or tetracycline sensitivities, at least under the experimental conditions applied in our study. These seemingly contradictory results between our studies and other previous results could be due to the fact that different set of *L. monocytogenes* strains were investigated in our study compared to those examined by Kremer et al. (2017). It can also not be ruled out that the observed differences in antibiotic susceptibility between pLMST6 transformed and naive strains could also have been genotype specific since *L. monocytogenes* examined in the two studies also belong to different STs. Based on the observations in our study, we, however, found in the context of different *L. monocytogenes* genetic backgrounds that the pLMST6 plasmid associated genes do not have a significant impact on susceptibility of this organism to antibiotics such as ampicillin and gentamicin that may be used for the treatment of listeriosis.

Recent studies from the Netherlands found an epidemiological association between pLMST6 plasmid presence and listerial meningitis with mostly unfavorable disease outcome due to ST6 *L. monocytogenes* strains (Kremer et al., 2017). Plasmid harboring ST6 isolates described did not only show reduced susceptibility to BC but also an increased tolerance to gentamicin and amoxicillin. These attributes might therefore contribute to increased virulence and severe disease outcomes in patients infected with pLMST6 harboring *L. monocytogenes* ST6 isolates. But besides these *emrC* related direct impacts on disinfectant and antibiotic resistance increasing virulence it remains plausible that the pLMST6 could also have an influence in *L. monocytogenes* pathogenicity through other plasmid encoded virulence factors. To examine this possibility, virulence was compared between two pLMST6 plasmid harboring *L. monocytogenes* strains (N12-0935 and N12-2082) and their corresponding plasmid cured variants revealing that virulence was increased in presence of this plasmid. In zebrafish embryos infected with pLMST6 plasmid harboring variants of *L. monocytogenes* N12-0935 and N12-2082 strains, a significant reduction in survival was observed when compared to those infected with corresponding plasmid cured variants of these two strains. Our results thus suggest that pLMST6 presence enhances the virulence of *L. monocytogenes* but the exact mechanisms of how this plasmid is involved in this process remain unknown. It must also be stressed that to determine if there is any correlation between pLMST6 plasmid and the strain’s potential to cause meningitis experiments using appropriate listeria meningitis model will be necessary in future.

## Conclusion

In conclusion, we have shown that the presence of pLMST6 plasmid is linked with BC tolerance in *L. monocytogenes* as supported through investigations conducted at genome, transcriptome and proteome levels. At genome level we could confirm that this plasmid is associated with conferring as well as mobilization of an increased BC tolerance phenotype in *L. monocytogenes* and *L. innocua*. A targeted analysis of transcripts and proteins derived from pLMST6 genes provided further molecular insights into the expression regulation of genes associated with this plasmid during *L. monocytogenes* response to BC stress. Our results have shown that besides protection against BC the pLMST6 plasmid also increases tolerance to other types of QAC based disinfectants as well as contribute to increased virulence potential in *L. monocytogenes* through as of yet unknown plasmid associated mechanisms. On the other hand, the direct role of the plasmid encoded EmrC efflux pump system in these observed BC tolerance phenotypes, however, will still need to be conclusively shown through future experiments that might include the generation and phenotypic characterization of an *emrC* deletion mutant of the pLMST6 plasmid. To the best of our knowledge, this is the first report combining and comparing genomic, transcriptomic and proteomic effects on sublethal concentrations of BC on the expression of pLMST6 associated genes in *L. monocytogenes*. Since QACs are commonly used in food processing environment, there is a growing concern that *L. monocytogenes* strains harboring pLMST6 plasmid will have survival advantages and lead to the persistence of this pathogen. Meanwhile from a food safety perspective, it is pivotal that we better understand how *L. monocytogenes* copes with sublethal concentrations of BC in the fight against this important food borne pathogen in order to prevent product recalls as well as human infection and listeriosis disease outbreaks.

## Data Availability

The datasets generated for this study can be found in NCBI, CP038642 and CP038643.

## Author Contributions

TT and AK designed the study. TT supervised the study. AK and AE performed the experiments. AK, TT, and RS wrote the manuscript.

## Conflict of Interest Statement

The authors declare that the research was conducted in the absence of any commercial or financial relationships that could be construed as a potential conflict of interest.

## References

[B1] Aase B. Sundheim G. Langsrud S. Rørvik L. M. (2000). Occurrence of and a possible mechanism for resistance to a quaternary ammonium compound in *Listeria monocytogenes*. *Int. J. Food Microbiol.* 62 57–63. 10.1016/S0168-1605(00)00357-3 11139022

[B2] Althaus D. Jermini M. Giannini P. Martinetti G. Reinholz D. Nüesch-Inderbinen M. (2017). Local outbreak of *Listeria monocytogenes* serotype 4b sequence type 6 due to contaminated meat pâté. *Foodborne Pathog. Dis.* 14 219–222. 10.1089/fpd.2016.2232 28379731

[B3] Bille J. Blanc D. S. Schmid H. Boubaker K. Baumgartner A. Siegrist H. H. (2005). Outbreak of human listeriosis associated with tomme cheese in northwest Switzerland, 2005. *Euro surveill.* 11 91–93. 16801693

[B4] Boguslawska J. Zycka-Krzesinska J. Wilcks A. Bardowski J. (2009). Intra- and interspecies conjugal transfer of Tn916-like elements from *Lactococcus lactis in vitro* and *in vivo*. *Appl. Environ. Microbiol.* 75 6352–6360. 10.1128/AEM.00470-09 19666731 PMC2753074

[B5] Chandramouli K. Qian P. Y. (2009). Proteomics: challenges, techniques and possibilities to overcome biological sample complexity. *Hum. Genomics Proteomics* 2009:239204. 10.4061/2009/239204 20948568 PMC2950283

[B6] Deng W. Li C. Xie J. (2013). The underling mechanism of bacterial TetR/AcrR family transcriptional repressors. *Cell. Signal.* 25 1608–1613. 10.1016/j.cellsig.2013.04.003 23602932

[B7] Dutta V. Elhanafi D. Kathariou S. (2013). Conservation and distribution of the benzalkonium chloride resistance cassette *bcrABC* in *Listeria monocytogenes*. *Appl. Environ. Microbiol.* 79 6067–6074. 10.1128/AEM.01751-13 23892748 PMC3811391

[B8] Ebner R. Stephan R. Althaus D. Brisse S. Maury M. Tasara T. (2015). Phenotypic and genotypic characteristics of *Listeria monocytogenes* strains isolated during 2011-2014 from different food matrices in Switzerland. *Food Control* 57 321–326. 10.1016/j.foodcont.2015.04.030

[B9] EFSA (2018). The European Union summary report on trends and sources of zoonoses, zoonotic agents and food-borne outbreaks in 2017. *EFSA J.* 16:5500 10.2903/j.efsa.2018.5500 PMC7009540 32625785

[B10] Elhanafi D. Dutta V. Kathariou S. (2010). Genetic characterization of plasmid-associated benzalkonium chloride resistance determinants in a *Listeria monocytogenes* strain from the 1998-1999 outbreak. *Appl. Environ. Microbiol.* 76 8231–8238. 10.1128/AEM.02056-10 20971860 PMC3008257

[B11] Eshwar A. K. Guldimann C. Oevermann A. Tasara T. (2017). Cold-shock domain family proteins (Csps) are involved in regulation of virulence, cellular aggregation, and flagella-based motility in *Listeria monocytogenes*. *Front. Cell. Infect. Microbiol.* 7:453. 10.3389/fcimb.2017.00453 29124040 PMC5662587

[B12] Fox E. M. Leonard N. Jordan K. (2011). Physiological and transcriptional characterization of persistent and nonpersistent *Listeria monocytogenes* isolates. *Appl. Environ. Microbiol.* 77 6559–6569. 10.1128/AEM.05529-11 21764947 PMC3187160

[B13] Goren M. G. Carmeli Y. Schwaber M. J. Chmelnitsky I. Schechner V. Navon-Venezia S. (2010). Transfer of carbapenem-resistant plasmid from *Klebsiella pneumoniae* ST258 to *Escherichia coli* in patient. *Emerg. Infect. Dis.* 16 1014–1017. 10.3201/eid1606.091671 20507761 PMC3086234

[B14] Haase J. K. Korkeala H. Didelot X. Lecuit M. Achtman M. (2014). The ubiquitous nature of *Listeria monocytogenes* clones: a large-scale multilocus sequence typing study. *Environ. Microbiol.* 16 405–416. 10.1111/1462-2920.12342 24274459

[B15] Hächler H. Marti G. Giannini P. Lehner A. Jost M. Beck J. (2013). Outbreak of listerosis due to imported cooked ham, Switzerland 2011. *Euro Surveill.* 18:20469. 23725774

[B16] Kelstrup C. D. Young C. Lavallee R. Nielsen M. L. Olsen J. V. (2012). Optimized fast and sensitive acquisition methods for shotgun proteomics on a quadrupole orbitrap mass spectrometer. *J. Proteome Res.* 11 3487–3497. 10.1021/pr3000249 22537090

[B17] Kocharunchitt C. King T. Gobius K. Bowman J. P. Ross T. (2012). Integrated transcriptomic and proteomic analysis of the physiological response of *Escherichia coli* O157:H7 Sakai to steady-state conditions of cold and water activity stress. *Mol. Cell. Proteomics* 11 M111.009019. 10.1074/mcp.M111.009019 22008207 PMC3270098

[B18] Kovacevic J. Ziegler J. Walecka-Zacharska E. Reimer A. Kitts D. D. Gilmour M. W. (2015). Tolerance of *Listeria monocytogenes* to quaternary ammonium sanitizers is mediated by a novel efflux pump encoded by EmrE. *Appl. Environ. Microbiol.* 82 939–953. 10.1128/AEM.03741-15 26590290 PMC4725271

[B19] Kremer P. H. Lees J. A. Koopmans M. M. Ferwerda B. Arends A. W. Feller M. M. (2017). Benzalkonium tolerance genes and outcome in *Listeria monocytogenes* meningitis. *Clin. Microbiol. Infect.* 23 265.e1–265.e7. 10.1016/j.cmi.2016.12.008 27998823 PMC5392494

[B20] Kües U. Stahl U. (1989). Replication of plasmids in Gram-negative bacteria. *Microbiol. Rev.* 53 491–516. 2687680 10.1128/mr.53.4.491-516.1989 PMC372750

[B21] Liu Y. Beyer A. Aebersold R. (2016). On the dependency of cellular protein levels on mRNA abundance. *Cell* 165 535–550. 10.1016/j.cell.2016.03.014 27104977

[B22] Lomovskaya O. Lewis K. (1992). Emr, an *Escherichia coli* locus for multi-drug resistance. *Proc. Natl. Acad. Sci.* 89 8938–8942. 10.1073/pnas.89.19.8938 1409590 PMC50039

[B23] Maertens de Noordhout C. Devleesschauwer B. Angulo F. J. Verbeke G. Haagsma J. Kirk M. (2014). The global burden of listeriosis: a systematic review and meta-analysis. *Lancet Infect. Dis.* 14 1073–1082. 10.1016/S1473-3099(14)70870-9 25241232 PMC4369580

[B24] Maury M. M. Tsai Y. H. Charlier C. Touchon M. Chenal-Francisque V. Leclercq A. (2016). Uncovering *Listeria monocytogenes* hypervirulence by harnessing its biodiversity. *Nat. Genet.* 48 308–313. 10.1038/ng.3501 26829754 PMC4768348

[B25] McDonnell G. Russell A. D. (1999). Antiseptics and disinfectants: activity, action, and resistance. *Clin. Microbiol. Rev.* 12 147–179. 10.1128/CMR.12.1.147 9880479 PMC88911

[B26] Meier A. B. Guldimann C. Markkula A. Pöntinen A. Korkeala H. Tasara T. (2017). Comparative phenotypic and genotypic analysis of Swiss and Finnish *Listeria monocytogenes* isolates with respect to benzalkonium chloride resistance. *Front. Microbiol.* 8:397. 10.3389/fmicb.2017.00397 28386248 PMC5362634

[B27] Mereghetti L. Quentin R. Marquet-van Der Mee N. Audurier A. (2000). Low sensitivity of *Listeria monocytogenes* to quaternary ammonium compounds. *Appl. Environ. Microbiol.* 66 5083–5086. 10.1128/AEM.66.11.5083-5086.2000 11055967 PMC92423

[B28] Michel C. J. (2012). Circular code motifs in transfer and 16S ribosomal RNAs: a possible translation code in genes. *Computat. Biol. Chem.* 34 24–37. 10.1016/j.compbiolchem.2011.10.002 22129773

[B29] Monk I. R. Gahan C. G. Hill C. (2008). Tools for functional postgenomic analysis of *Listeria monocytogenes*. *Appl. Environ. Microbiol.* 74 3921–3934. 10.1128/AEM.00314-08 18441118 PMC2446514

[B30] Morente E. O. Fernández-Fuentes M. A. Burgos M. J. G. Abriouel H. Pulido R. P. Gálvez A. (2013). Biocide tolerance in bacteria. *Int. J. Food Microbiol.* 162 13–25. 10.1016/j.ijfoodmicro.2012.12.028 23340387

[B31] Müller A. Rychli K. Muhterem-Uyar M. Zaiser A. Stessl B. Guinane C. M. (2013). Tn6188 - a novel transposon in *Listeria monocytogenes* responsible for tolerance to benzalkonium chloride. *PLoS One* 8:e76835. 10.1371/journal.pone.0076835 24098567 PMC3788773

[B32] Murray E. G. D. Webb R. A. Swann M. B. R. (1926). A disease of rabbits characterised by a large mononuclear leucocytosis, caused by a hitherto undescribed bacillus *Bacterium monocytogenes* (n.sp.). *J. Pathol. Bacteriol.* 29 407–439. 10.1002/path.1700290409

[B33] Orsi R. H. den Bakker H. C. Wiedmann M. (2011). *Listeria monocytogenes* lineages: genomics, evolution, ecology, and phenotypic characteristics. *Int. J. Med. Microbiol.* 301 79–96. 10.1016/j.ijmm.2010.05.002 20708964

[B34] Paulsen I. T. Skurray R. A. Tam T. Saier J. R. Turner R. J. Weiner J. H. (1996). The SMR family: a novel family of multidrug efflux proteins involved with the efflux of lipophilic drugs. *Mol. Microbiol.* 19 1167–1175. 10.1111/j.1365-2958.1996.tb02462.x 8730859

[B35] Ramos J. L. Martınez-Bueno M. Molina-Henares A. J. Teran W. Watanabe K. Zhang X. (2005). The TetR family of transcriptional repressors. *Microbiol. Mol. Biol. Rev.* 69 326–356. 10.1128/MMBR.69.2.326 15944459 PMC1197418

[B36] Schwartz K. T. Carleton J. D. Quillin S. J. Rollins S. D. Portnoy D. A. Leber J. H. (2012). Hyperinduction of host beta interferon by a *Listeria monocytogenes* strain naturally overexpressing the multidrug efflux pump MdrT. *Infect. Immun.* 80 1537–1545. 10.1128/IAI.06286-11 22290148 PMC3318417

[B37] Smith M. A. Bidochka M. J. (1998). Bacterial fitness and plasmid loss: the importance of culture conditions and plasmid size. *Can. J. Microbiol.* 44 351–355. 10.1139/w98-020 9674107

[B38] Tasara T. Stephan R. (2007). Evaluation of housekeeping genes in *Listeria monocytogenes* as potential internal control references for normalizing mRNA expression levels in stress adaptation models using real-time PCR. *FEMS Microbiol. Lett.* 269 265–272. 10.1111/j.1574-6968.2007.00633.x 17263845

[B39] Tauxe R. V. Cavanagh T. R. Cohen M. L. (1989). Interspecies gene transfer *in vivo* producing an outbreak of multiply resistant Shigellosis. *J. Infect. Dis.* 160 1067–1070. 10.1093/infdis/160.6.1067 2685126

[B40] To M. S. Favrin S. Romanova N. Griffiths M. W. (2002). Postadaptational resistance to benzalkonium chloride and subsequent physicochemical modifications of *Listeria monocytogenes*. *Appl. Environ. Microbiol.* 68 5258–5264. 10.1128/AM.68.11.5258 12406712 PMC129876

[B41] Valenzuela M. S. Ikpeazu E. V. Siddiqui K. A. (1996). *E. coli* growth inhibition by a high copy number derivative of plasmid pbr322. *Biochem. Biophys. Res. Commun.* 219 876–883. 10.1006/bbrc.1996.0339 8645273

[B42] Weinmaier T. Riesing M. Rattei T. Bille J. Arguedas-Villa C. Stephan R. (2013). Complete genome sequence of *Listeria monocytogenes* LL195, a serotype 4b strain from the 1983 – 1987 listeriosis epidemic in Switzerland. *Genome Announc.* 1 8–9. 10.1128/genomeA.00152-12 23405339 PMC3569335

[B43] Xu D. Li T. Zahid M. S. Yamasaki S. Shi L. Li J. R. (2014). Benzalkonium chloride and heavy-metal tolerance in *Listeria monocytogenes* from retail foods. *Int. J. Food Microbiol.* 190 24–30. 10.1016/j.ijfoodmicro.2014.08.017 25173916

[B44] Zeevi M. K. Friedman S. Shaham S. Herskovits A. A. Shafir N. S. Sigal N. (2013). *Listeria monocytogenes* multidrug resistance transporters and cyclic di-AMP, which contribute to type I interferon induction, play a role in cell wall stress. *J. Bacteriol.* 195 5250–5261. 10.1128/jb.00794-13 24056102 PMC3837957

